# Time-Released Black Phosphorus Hydrogel Accelerates Myocardial Repairing through Antioxidant and Motivates Macrophage Polarization Properties

**DOI:** 10.34133/bmr.0029

**Published:** 2024-05-08

**Authors:** Jiahui Zhang, Di Sun, Yuhan Liao, Bingxin Cao, Ran Gao, Zhuanglin Zeng, Chuansheng Zheng, Yumiao Wei, Xiaopeng Guo

**Affiliations:** ^1^ Department of Cardiology, Union Hospital, Tongji Medical College, Huazhong University of Science and Technology, Wuhan 430022, China.; ^2^ Hubei Key Laboratory of Biological Targeted Therapy, Union Hospital, Tongji Medical College, Huazhong University of Science and Technology, Wuhan 430022, China.; ^3^ Hubei Engineering Research Center for Immunological Diagnosis and Therapy of Cardiovascular Diseases, Union Hospital, Tongji Medical College, Huazhong University of Science and Technology, Wuhan 430022, China.; ^4^ Department of Plastic Surgery, Union Hospital, Tongji Medical College, Huazhong University of Science and Technology, Wuhan 430022, China.; ^5^ Cardiac Surgery, Union Hospital, Tongji Medical College, Huazhong University of Science and Technology, Wuhan 430022, China.; ^6^ Department of Radiology, Union Hospital, Tongji Medical College, Huazhong University of Science and Technology, Wuhan 430022, China.

## Abstract

The improvement of the myocardial microenvironment largely determines the prognosis of myocardial infarction (MI). After MI, early removal of excessive reactive oxygen species (ROS) in the microenvironment can alleviate oxidative stress injury and promote M2 phenotype polarization of macrophages, which is important for advocating myocardial repair. In this study, we combined traditional natural hydrogel materials chitosan (CS) and gelatin (Gel) to encapsulate polydopamine-modified black phosphorus nanosheets (BP@PDA). We designed an injectable composite gel (CS–Gel–BP@PDA) with a time-released ability to achieve in situ sustained-release BP@PDA in the area of MI. Utilizing the inflammation inhibition ability of CS-Gel itself and the high reactive activity of BP@PDA with ROS, continuous improvement of infarct microenvironment and myocardial repair were achieved. The studies in vivo revealed that, compared with the saline group, CS-Gel-BP@PDA group had alleviated myocardial fibrosis and infarct size and importantly improved cardiac function. Immunofluorescence results showed that the ROS level and inflammatory response in the microenvironment of the CS–Gel–BP@PDA group were decreased. In conclusion, our study demonstrated the time-released ability, antioxidative stress activity and macrophage polarization modulation of the novel composite hydrogel CS-Gel-BP@PDA, which provides inspiration for novel therapeutic modalities for MI.

## Introduction

Myocardial infarction (MI), the most common cause of heart failure, is a life-threatening condition [1]. In clinical treatment, MI is usually treated by restoration of reperfusion, including interventional surgery and thrombolytic drugs. However, mortality and readmission rates for heart failure due to current clinical modalities remain high [2,3]. This is mainly attributed to the inability to adequately treat the oxidative stress and inflammatory response already occurring in the infarct microenvironment. Repair of cardiomyocytes at the infarct site requires consideration of many factors such as oxidative stress, inflammatory response, and hypoxia–ischemia [4]. Therefore, therapeutic strategies that can attenuate oxidative stress and inflammatory responses in the infarct microenvironment early in MI are warranted. After MI, the microenvironment of ischemia–hypoxia leads to the impairment of mitochondrial function in myocardial cells and the release of a large number of reactive oxygen species (ROS). ROS leads to irreversible damage in cardiomyocytes by triggering cytochrome c and cysteine protease signaling pathways [5,6]. In this process, ROS can produce chemokines and proinflammatory factors by activating the nuclear factor κB (NF-κB) signaling pathway. ROS acts together with a large number of proinflammatory factors secreted by macrophages to induce an inflammatory response at the infarct site [7]. Macrophages are the primary response cells of the immune system that infiltrate infarcted myocardium for cardiac repair. In the early stage of MI, the infiltration of M1 phenotype macrophages promotes the phagocytosis of necrotic tissues and neutrophils, leading to an inflammatory response. Subsequently, M2 phenotype macrophages dominate tissue repair and reconstruction during the resolution phase of inflammation [8,9]. In this process, macrophage phenotype and oxidative stress regulation are critical for cardiac recovery. Consequently, a combination of ROS depletion in the infarct microenvironment to prevent oxidative stress and macrophage polarization to the M2 phenotype may be an ideal strategy to improve the prognosis of MI.

Biomaterials such as graphene oxide and fullerenol promote myocardial repair by eliminating ROS. Antioxidant drugs (glutathione and ascorbic acid) or anti-inflammatory plasmids (interleukin-4 [IL-4] and IL-10) are also used to modulate the immune microenvironment in MI [10]. In these studies, the restoration of cardiac function relies on the combination of complex biomaterials with stem cells, drugs, or genes. This may be due to the fact that their modulation of ROS is not sufficient for MI therapy [11,12]. Combined use may enhance therapeutic effects, but there are potential safety issues with degradability, biocompatibility, and clearance. Therefore, there is a need for a safe and effective biomaterial that simultaneously exerts ROS scavenging and promotes macrophage M2 phenotype polarization for myocardial repair in the MI microenvironment. Black phosphorus nanosheets (BPNSs) are a kind of nanomaterials with 2-dimensional layered structure. Studies have shown that BPNSs have potent ROS-responsive activity, which can effectively scavenge ROS and thus promote macrophage polarization toward the M2 phenotype and inhibit the activation of the subsequent inflammatory response chain. Therefore, BPNSs have been widely used for diabetic skin wounds, bone regeneration, and neurovascular regeneration [13,14]. We reasonably hypothesized that polydopamine (PDA)-modified BPNSs could efficiently and adequately scavenge ROS in the MI microenvironment, attenuate oxidative stress, inhibit inflammatory responses, improve the cardiac microenvironment, and repair damaged myocardium.

In recent years, hydrogels have been widely studied as novel biomaterials with the advantages of injectability, high drug loading rate, and degradability [15]. Chitosan (CS) is a natural polymer consisting of partially acetylated (1-4)-2-amino-2-deoxy-d-glucan, which is widely used in tissue engineering due to its biocompatibility, biodegradability, as well as antimicrobial and immunogenic properties [16,17]. Previous studies have shown that CS oligosaccharides, a degradation product of CS, are capable of resisting oxidative stress and inhibiting inflammatory responses, which is achieved through the activation of intracellular antioxidant enzyme production and inhibition of the NF-κB signaling pathway [18–21]. However, the therapeutic activity of CS decreases with an increase in its molecular weight concentration due to the presence of strong inter- and intramolecular hydrogen bonds between CS molecules. Therefore, it is necessary to design CS-based hydrogels with high molecular weights while still maintaining good antioxidant capacity. Gelatin (Gel) is a protein derived from the hydrolysis of collagen, and its biocompatibility and degradability have been confirmed by numerous studies [22–24]. It has been shown that the composite hydrogel formed by CS and Gel is able to overcome the hydrophobicity of CS, which is favorable for in vivo applications. Importantly, Gel cross-linked with CS can break the hydrogen bond between amino and hydroxyl groups in CS, which facilitates CS to exert more powerful antioxidant effects [25–27]. Therefore, we designed an injectable hydrogel (CS-Gel) with controlled-release capability by combining CS with Gel to encapsulate PDA-modified BPNSs (BP@PDA) to achieve efficient drug release accumulation in the infarct region, which lays a good foundation for the subsequent biotherapeutic applications.

**Fig. 1.**
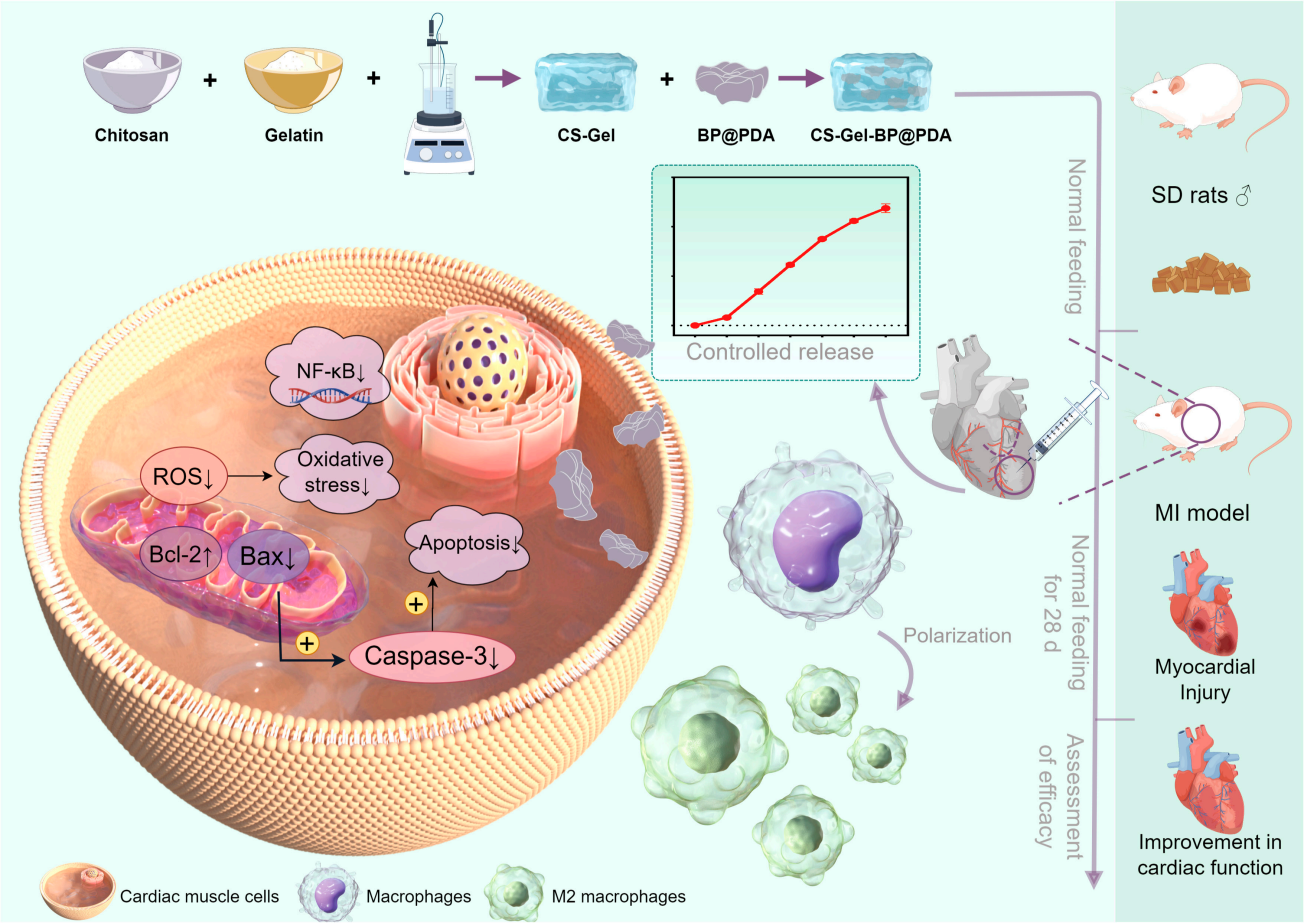
Schematic diagram of synthesis and action mechanism of CS–Gel–BP@PDA.

In this study, we injected a composite hydrogel (CS-Gel-BP@PDA) in situ within the myocardium, which was able to target and continuously deliver BP@PDA to modulate oxidative stress and macrophage phenotypes in the MI microenvironment, leading to efficient myocardial repair (Fig. 1). CS-Gel-BP@PDA composite hydrogel is expected to scavenge the overloaded ROS in the MI microenvironment and promote macrophage M2 phenotypic polarization to rescue dying myocardium, reduce infarct size, inhibit infarct progression, and ultimately improve cardiac function through the sustained release of BP@PDA while providing mechanical support. We believe that this study will fill the gap of MI microenvironment therapeutics and provide new ideas for clinical treatment of MI.

## Materials and Methods

### Preparation of BPNSs and BP@PDA

Black phosphorus block precursors were prepressed at room temperature, followed by rapid heating to 1,000 °C. BPNSs were subsequently prepared using electrochemical peeling. Black phosphorus tablets were prepared in propylene carbonate and tetrabutylammonium hexafluorophosphate. The black phosphorus crystal was used as the cathode, and the platinum sheet was used as the anode. The experiment proceeded at a constant voltage of −5 V. Subsequently, the peeled black phosphorus sheet was dispersed into anhydrous acetone by sonication for 1 h. Thicker BP slices were removed by centrifugation at 1,500 rpm for 10 min. Next, the BPNSs were collected by centrifugation at 5,000 rpm for 10 min. After removing the supernatant, BPNSs were dispersed into ultrapure water. BPNSs were then added to an alkaline solution with pH = 8.5, 100 μl of aqueous dopamine hydrochloride (50 mg ml^−1^) was added, and the mixture was stirred for 5 h in the dark. Subsequently, pBP@Mg was obtained by centrifugation at 5,000 rpm for 15 min.

### Preparation of hydrogel

The hydrogel was synthesized as previously reported [28]. CS 2 w/v% was dissolved in 10 ml of 1 wt% acetic acid and stirred for 2 h to obtain a CS solution. Gel solution (2 wt%) was prepared by dissolving the Gel in sterile water at 50 °C until it was completely dissolved and then filtering through a sheet of 100-mesh filter paper. Subsequently, the CS and Gel solutions were mixed by vigorous stirring, 0.25 wt% glutaraldehyde was added as a cross-linking agent, and the CS–Gel solution was formed by stirring at 50 °C for 12 h.

### Characterization of BPNSs and BP@PDA

Topography and surface elemental scanning were tested using a transmission electron microscope (JEOL, JEM-F200, Japan). An atomic force microscope (AFM) (Bruker, Dimension Icon, Germany) was used to test the thickness of the nanomaterials. Crystallinity was tested by x-ray diffraction (XRD) (X’Pert PRO MPD). Raman spectra were tested using a Raman microscopy device (Horiba LabRAM HR Evolution, Japan) with a 532-nm laser excitation. Fourier transform infrared spectroscopy (FTIR) (Nicolet iS 10) detected the information of chemical bonds in the wave-number range of 500 to 4,000 cm^−1^. Zeta potential (Malvern Zetasizer Nano ZS90) was used to detect potential conditions. Ultraviolet-visible (UV-Vis) spectroscopy (3600 UV-Vis spectrometer, Shimadzu, Japan) tested the light absorption characteristics.

### Characterization of CS–Gel and CS–Gel–BP@PDA

The microstructure of the cross-sections of the hydrogel was observed via a scanning electron microscope (ZEISS, GeminiSEM 300, Germany). The CS–Gel and CS–Gel–BP@PDA were freeze-dried, and then the cross-sections of the hydrogel samples were coated with gold to improve their electrical conductivity.

### Rheological testing

The modulus was tested using a rheometer (Haake Mars60, Germany). The hydrogel was placed on a 20-mm parallel plate at room temperature using a fixed strain of 0.5%.

### Swelling experiment

The equilibrium swelling ratio was used to test the swelling properties. The initial weight of the hydrogel was *W*_0_. The hydrogel was soaked in phosphate-buffered saline at 37 °C, removed after 24 h, and dried the *W_t_* was measured again. The swelling ratio was calculated using the formula:

$$\text{Swelling ratio}\;\left(g/g\right)=\left(W_{t}-W_{0}\right)/W_{0}$$

### CS–Gel–BP@PDA sustained-release capability

The release of P element from degradable BPNSs in the CS–Gel–BP@PDA hydrogel scaffold was analyzed using inductively coupled plasma atomic emission spectroscopy. Each hydrogel sample (0.5 ml) was immersed in 6 ml of normal saline and incubated at 37 °C for up to 21 d.

### ROS scavenging ability of CS–Gel–BP@PDA

The antioxidant activity of the composite hydrogel was evaluated by scavenging 2,2-diphenyl-1-(2,4,6-trinitrobenzene) hydrazine (DPPH) free radicals. A total of 500 μl of DPPH ethanol solution (0.04 mg ml^−1^) was added to the surface of different hydrogels and placed at room temperature in the dark for 2 h. The absorbance of the supernatant at 515 nm was measured using a UV-Vis spectrophotometer. The following equation was used:

$$\text{Scavenging effect}=\frac{A_{\text{control}}-A_{\text{sample}}}{A_{\text{control}}}\times 100\%$$

The total antioxidant capacity of the composite hydrogel was measured using the ABTS (2,2-azino-bis (3-ethylbenzo-thiazoline-6-sulfonic acid diammonium salt) method. ABTS detection solution (500 μl) was added to the surface of the different hydrogels and allowed to stand in the dark at room temperature for 6 min. The absorbance of the supernatant was determined at 405 nm. The following equation was used:

$$\text{Scavenging effect}=\frac{A_{\text{control}}-A_{\text{sample}}}{A_{\text{control}}}\times 100\%$$

### In vitro cell culture

H9C2 cells were cultured at 37 °C in an incubator containing 5% CO_2_, and nutrients were obtained from a high-glucose medium containing 10% fetal bovine serum and 1% double antibody.

### Cell viability and biosafety assay

H9C2 cells were seeded at a density of 5,000 cells per well in 96-well culture plates lined with different hydrogels. When the cells reached 70% to 80% density, 0.1 mM H_2_O_2_ was added and treated for 24 h. Subsequently, 10 μl of Cell Counting Kit-8 (CCK-8) solution (Solarbio, China) was added to each well and treated for 2 h. The absorbance was measured at a wavelength of 450 nm.

### Live/dead cell viability assay

Cell viability was determined using calcein acetoxymethyl ester (calcein-AM) staining. The cells were incubated with 10% cell medium volume of calcein-AM for 30 min and washed twice with phosphate-buffered saline. The cells were observed via fluorescence microscopy.

### Evaluation of ROS scavenging capacity

Dihydroethidium (DHE) and 2,7-dichlorodihydrofluorescein diacetate (DCFH-DA) were used to detect the level of intracellular ROS. The cells were treated with 0.1 mM H_2_O_2_ for 12 h to induce oxidative stress. Next, DHE or DCFH-DA was added and incubated at 37 °C in the dark for 30 min. The cells were observed under a fluorescence microscope. The relative fluorescence intensities of DHE and DCFH-DA were calculated using ImageJ Plus 6.0.

### Mitochondrial membrane potential detection

For this, 0.1 mM H_2_O_2_ was added and treated for 12 h in the dark, incubated at 37 °C for 20 min, and washed twice with 1× buffer after completion of the treatment. The cells were observed under a fluorescence microscope.

### Rat MI and hydrogel injection model

Male Sprague–Dawley rats (7 wk old, 240 to 260 g) were purchased from Vital River (Beijing, China). The rats were mechanically ventilated and anesthetized with 10% chloral hydrate. The heart was exposed through a left lateral thoracotomy, and MI was induced by ligating the left anterior descending coronary artery with 8–0 polypropylene sutures. Hydrogel (20 μl per site) was subsequently injected into the infarct area and 5 sites surrounding it. Animal experimental procedures were approved by the Institutional Animal Care and Use Committee (IACUC), Huazhong University of Science and Technology (IACUC Number: 3440).

### Rat electrocardiogram and echocardiography

Vevo 2100 ultrasound imaging system was used to evaluate left ventricular (LV) function in each group. Electrocardiography was performed 24 h after the MI model was established. Electrocardiography was also performed 28 d later. A short-axis view was positioned at the level of the papillary muscles using transthoracic 2-dimensional ultrasound-guided M-mode. Cardiac function was measured as the mean of 3 consecutive cardiac cycles.

### Histomorphological analysis

The rats were euthanized 28 d after surgery. The hearts were quickly excised and fixed in paraformaldehyde. Infarct size and LV wall thickness in the MI zone were detected by Masson staining (Servicebio, Wuhan, China). Hematoxylin and eosin (H&E) staining (Servicebio, Wuhan, China) was used to observe the tissue morphology of the MI area and toxicity in other organs.

### Immunofluorescence assay

Heart samples were taken from each group, fixed in 4% (w/v) formaldehyde, paraffin-embedded, and sectioned. DHE immunofluorescence staining was used to detect ROS levels in frozen sections. Sections were sequentially incubated with primary antibody, secondary antibody, and 4′,6-diamidino-2-phenylindole and then examined under a fluorescence microscope. Caspase-3 immunofluorescence staining was used to assess cardiomyocyte apoptosis. Tumor necrosis factor-α (TNF-α) and IL-10 immunofluorescence staining was used to assess inflammatory response. CD86/CD206 immunofluorescence colocalization was used to detect macrophage phenotype. Images were obtained using a fluorescence microscope (Apo Tome, Zeiss, Germany). Positive expression of relevant proteins in each region was quantified using ImageJ.

### Western blot analysis

Heart tissue was ground with grinding beads and sonicated for 5 cycles. A loading buffer was added after protein concentration was measured by the bicinchoninic acid method and then boiled and stored at −20 °C. Electrophoresis was performed using sodium dodecyl sulfate-polyacrylamide gel electrophoresis. The sample was subsequently transferred to a polyvinylidene difluoride membrane. The primary and secondary antibodies were incubated using an enhanced chemiluminescence hypersensitive luminescent solution (Servicebio, Wuhan, China) and imaged with a chemiluminescence apparatus.

### Statistical analysis

All data are expressed herein as mean ± standard deviation (*n* = 3). One-way analysis of variance was performed using GraphPad Prism 8.0, and Student *t* test was also performed. *P* value < 0.05 was used as the cutoff value for statistical significance.

## Results

### Synthesis and characterization of BPNSs and BP@PDA nanosheets

**Fig. 2.**
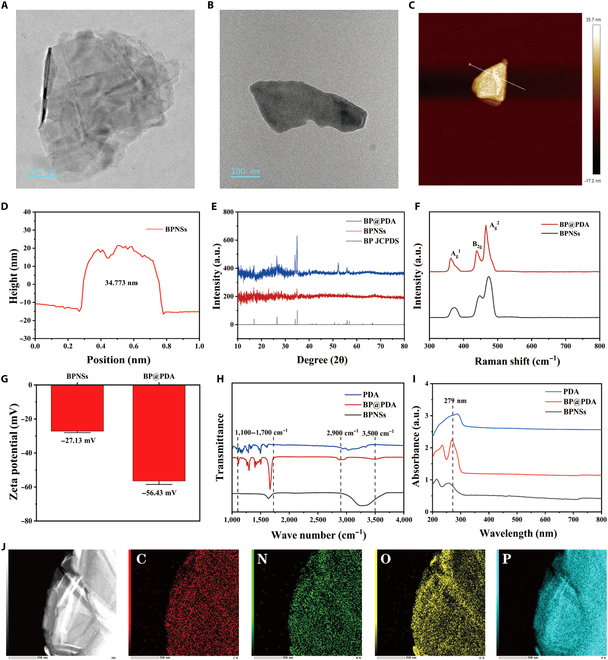
Characterization of BPNSs and BP@PDA. (A) Transmission electron microscopy of BPNSs. (B) Transmission electron microscopy of BP@PDA. (C) Atomic force microscopy of BPNSs and (D) thickness measurements. (E) Detection of XRD diffraction peaks of BPNSs and BP@PDA, compared with the standard black phosphorus block. (F) Absorption peaks of Raman spectra of BPNSs and BP@PDA. (G) Zeta potential detection of BPNSs and BP@PDA. (H) FTIR of BPNSs, PDA, and BP@PDA. (I) UV-Vis detection of BPNSs, PDA, and BP@PDA. (J) STEM image and STEM-EDX elemental mapping images of BP@PDA.

After the BPNSs were prepared, the BPNSs and PDA were added to a NaOH solution at pH = 8.5 in the dark and mixed thoroughly with magnetic stirring for 5 h to obtain BP@PDA. Scanning electron microscope and scanning transmission electron microscopy–energy-dispersive x-ray spectroscopy (STEM-EDX) inspection revealed that the sizes of the BPNSs and BP@PDA were about 300 to 400 nm, and the edges showed a typical 2-dimensional lamellar structure (Fig. 2A and B). C, N, O, and P elements are present on the surface of BP@PDA, and the various elements are uniformly distributed (Fig. 2J). Subsequently, the morphology and thickness of the BPNSs were examined by an AFM (Fig. 2C and D). The AFM showed that the maximum thickness of the BPNSs was 34.773 nm, and the BPNSs had a typical 2-dimensional lamellar structure. Next, the crystallization of BPNSs and BP@PDA was analyzed via XRD, and the absorption peaks of BPNSs and BP@PDA coincided well with those of the standard black phosphorus block (Fig. 2E). This indicates that the crystallinity of BPNSs and BP@PDA prepared by us did not change the crystal characteristics of black phosphorus and exhibited good crystallinity.

Next, we attempted to ascertain whether PDA modified BPN successfully. Raman spectroscopic detection of both BPNSs and BP@PDA showed the typical 3 absorption peaks, with the characteristic peaks near 363.8, 440.3, and 465.7 cm^−1^ being A_g_^1^, B_2g_, and A_g_^2^, respectively (Fig. 2F). This demonstrated that PDA modification did not alter the structural changes of BPNSs. The zeta potential proved that PDA was successfully modified (Fig. 2G). PDA was negatively charged, and after PDA modification, the zeta potential of BPNSs decreased from −27.13 to −56.43 mV. Zeta potential of BP@PDA was lower compared to that of bare BPNSs. Next, we examined the FTIR and UV-Vis spectra (Fig. 2H and I). FTIR results showed that the aromatic ring and the C–N bond in PDA caused BP@PDA to have a series of absorption bands between 1,200 and 1,600 cm^−1^. The 2 new absorption bands at 2,900 and 3,600 cm^−1^ were ascribed to C–H and N–H stretching vibrations in the PDA. UV-Vis spectroscopy results showed that BP@PDA had a strong light absorption at 279 nm, which was due to PDA. The above results further showed that PDA was successfully modified on the surface of BPNSs and tightly bound to BPNSs.

### Synthesis and characterization of the composite hydrogel

**Fig. 3.**
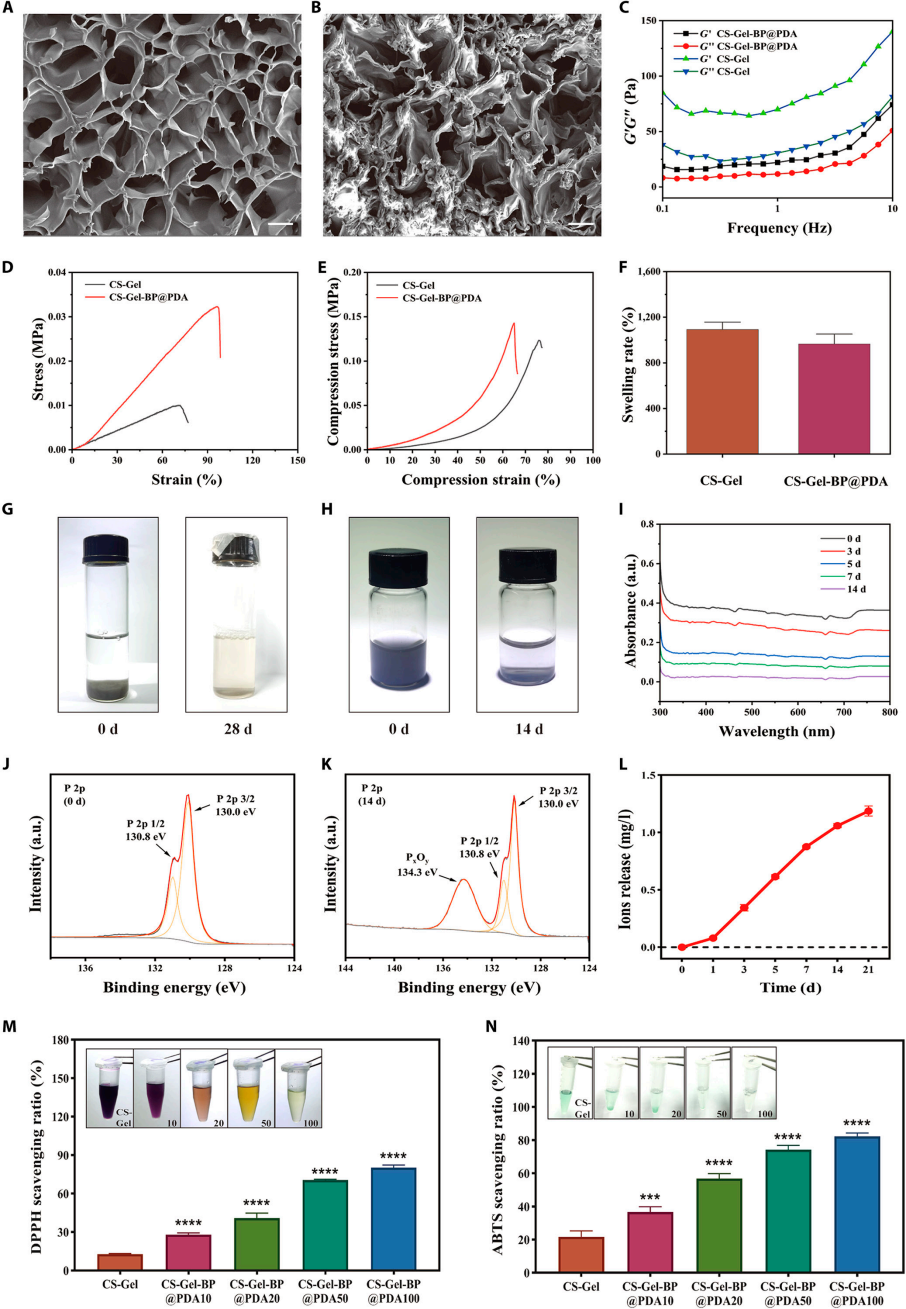
Characterization of the composite hydrogel. Representative scanning electron microscopy images of (A) CS-Gel and (B) CS-Gel-BP@PDA. (C) Rheological property test of CS-Gel and CS-Gel-BP@PDA. (D) Tensile-strain curves of CS-Gel and CS-Gel-BP@PDA. (E) Compression-strain curves of CS-Gel and CS-Gel-BP@PDA. (F) Dissolution performance test of CS-Gel and CS-Gel-BP@PDA. (G) Digital photographs of natural degradability of CS-Gel-BP@PDA. (H) Digital photographs of the degradability of BP@PDA. (I) UV-Vis spectra of supernatants of BP@PDA in H_2_O_2_ solution on days 0, 3, 5, 7, and 14. (J) X-ray photoelectron spectroscopy detection of supernatants of BP@PDA in H_2_O_2_ solution on day 0 and (K) day 14. (L) Composite hydrogel P-element release capacity curves. (M) DPPH radical scavenging ability of CS-Gel with different concentrations of CS-Gel-BP@PDA. (N) ABTS radical scavenging ability of CS-Gel with different concentrations of CS-Gel-BP@PDA. (Scale bar: 10 µm. *P < 0.05, **P < 0.01, ***P < 0.001, ****P < 0.0001)

CS–Gel was obtained by mixing CS and Gel, followed by adding cross-linking agent glutaraldehyde. CS–Gel–BP@PDA composite hydrogel was obtained by adding BP@PDA to the CS solution and then mixing it with Gel. After freeze drying, the cross-sectional morphology of the hydrogel was observed by scanning electron microscopy (Fig. 3A and B). All hydrogels have a loose porous structure with pore sizes ranging from 20 to 100 μm, which is essential for cell adhesion and nutrient exchange. Next, to elucidate the viscoelasticity of the hydrogels, the rheological properties of the hydrogels were measured in a certain frequency range (0.1 to 10 Hz) using a frequency scanning method (Fig. 3C). The 2 groups of hydrogels exhibited similar nonlinear rheological behavior. The G’ of the hydrogel was greater than G” in each frequency range, and the 3-dimensional network structure of the composite hydrogel showed good stability. In order to apply the composite hydrogel to the infarction site, CS-Gel-BP@PDA must have suitable mechanical characteristics. Therefore, we examined the tensile-strain curve and compressive-strain curve of both CS-Gel and CS-Gel-BP@PDA hydrogels (Fig. 3D and E and Fig. S1). The Young’s moduli obtained from the tensile-strain curves were 0.75 ± 0.059 MPa and 1.03 ± 0.01 MPa, respectively. The Young,s moduli obtained from the compression-strain curves were 26.67 ± 5.78 KPa and 36.67 ± 5.78 KPa, respectively. The Young’s moduli of both were similar to those of the natural myocardium, and CS-Gel-BP@PDA was closer [29]. This shows that the addition of BP@PDA resulted in better mechanical properties of the hydrogel.

Swelling property is a crucial characteristic of hydrogels. Good swelling properties can ensure the adequate exchange of oxygen and nutrients (Fig. 3F and Fig. S2). The dissolution rate of CS-Gel was 1,092.33%, while that of CS-Gel-BP@PDA was 966.33%, which was not significantly different from each other. The dissolution rate was slightly lower in the CS-Gel-BP@PDA group compared to CS-Gel. The combination of CS–Gel and BP@PDA likely changed the pore size of the hydrogel. As a result, CS–Gel–BP@PDA showed good material exchangeability. We further evaluated the biodegradability of the composite hydrogels in vitro (Fig. 3G and Fig. S3). After storage at 37 °C for 28 d, CS–Gel–BP@PDA was almost completely degraded, and the encapsulated BP@PDA was released into the solution. Subsequently, we placed the BP@PDA nanosheets into H_2_O_2_ solution to further investigate their degradability in the ROS environment (Fig. 3H and Fig. S4). After 14 d, the BP@PDA solution was importantly clarified compared to day 0. Its absorbance profile decreased importantly by UV-Vis spectrum detection (Fig. 3I). The degradability of BP@PDA was quantitatively monitored using light absorption at 450 nm as a reference point. After 14 d, the absorbance of BP@PDA decreased by 93.0%. The degradation of BP@PDA produced phosphorus oxides. Therefore, the supernatants from days 0 and 14 were collected to detect PxOy in them. The x-ray photoelectron spectroscopy assay showed that the content increased with time and the degradation of BP@PDA increased, with a distinct PxOy absorption peak at 134.3 eV (Fig. 3J and K).

To achieve in situ myocardial injection and maintain firm adhesion in heart tissue, the adhesive and injectability of the composite hydrogels were tested (Figs. S5 and S6). At room temperature, the composite hydrogels could be extruded through a 1-ml syringe to form stable long strip and mass structures (Movie S1). By applying 500 μl of CS–Gel–BP@PDA to your finger, the Eppendorf tube containing 1 ml of liquid could be easily adhered. In addition, to ensure the long-term controlled release of CS–Gel–BP@PDA, we tested the release curve of the P element (Fig. 3L). The release rate of P element was fast in the first 7 d and then gradually slowed down. On day 21, there was still P-element release, indicating that the composite hydrogel system had the ability to control release and could meet the needs of treating pathophysiological changes in MI for a long time.

### The antioxidant capacity of CS–Gel–BP@PDA

To evaluate the antioxidant capacity of the composite hydrogel, hydrogels containing different concentrations of BP@PDA were synthesized. We used CS–Gel–BP@PDA10, CS–Gel–BP@PDA20, CS–Gel–BP@PDA50, and CS–Gel–BP@PDA100 to represent BP@PDA composite hydrogels with concentrations of 10, 20, 50, and 100 μg ml^−1^, respectively. The antioxidant properties of composite hydrogels with different concentrations were evaluated by DPPH assay kit (Fig. 3M). DPPH radical is a kind of stable nitrogen-centered free radical. Its ethanol solution is dark purple and has strong absorption at 515 nm. In the presence of antioxidants, DPPH free radicals are removed, the color of the solution becomes lighter, and the absorbance at 515 nm decreases. The results show that the antioxidant performance of the hydrogel with the same volume is more significant with the increase of BP@PDA concentration. CS–Gel–BP@PDA100 achieved a free radical scavenging rate of 80.0%, an 84.0% improvement over hydrogel CS-Gel.

Next, we further assessed the total antioxidant capacity of the composites using the ABTS free radical scavenging capacity assay kit (Fig. 3N). ABTS^+^ was oxidized into green ABTS under the action of appropriate oxidants, with strong absorption at 405 nm. In the presence of antioxidants, the production of ABTS^+^ was inhibited, and the color of the solution became lighter. The clearance rate of CS–Gel–BP@PDA100 reached 82%, and the solution appeared almost colorless. Compared with CS-Gel, the ABTS radical scavenging rates of hydrogels containing different concentrations of BP@PDA were enhanced by 40.9%, 61.8%, 70.8%, and 73.7%, respectively. The above 2 groups of experiments proved that with the increase in BP@PDA concentration, the antioxidant ability of CS–Gel–BP@PDA was enhanced, and the highest free radical scavenging rate was obtained at CS–Gel–BP@PDA100.

### CS–Gel–BP@PDA ability to scour ROS in vitro

**Fig. 4.**
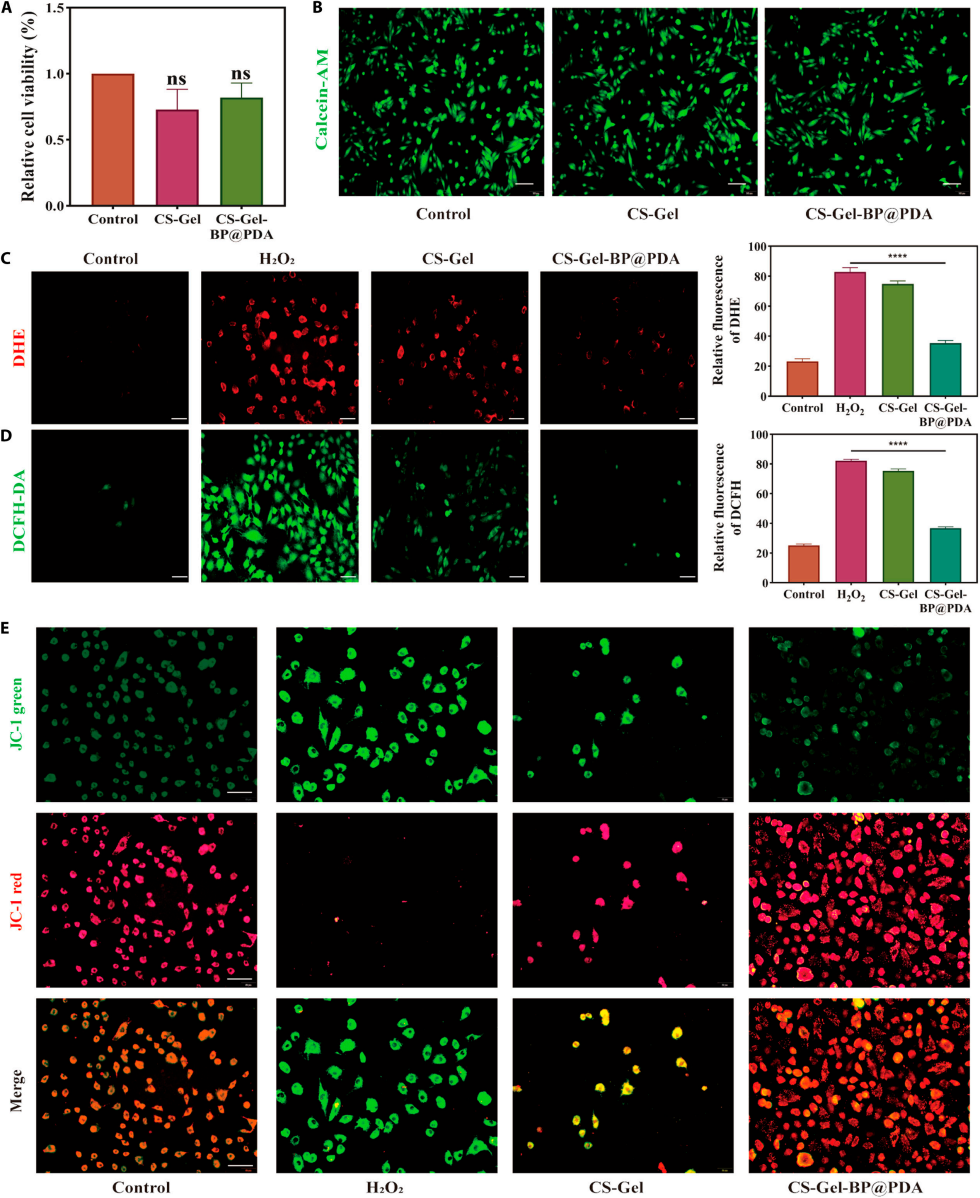
Antioxidant capacity of the composite hydrogel in vitro. (A) CCK-8 was used to detect the relative activity of H9C2 in different treatment groups. (B) Calcein-AM staining on CS–Gel and CS–Gel–BP@PDA after 24 h of culture. (C) DHE fluorescence staining and quantitative statistical analysis of different treatment groups. (D) DCFH-DA fluorescence staining and quantitative statistical analysis of different treatment groups. (E) JC-1 fluorescent staining of different treatment groups. (Scale bar: 50 µm. *P < 0.05, **P < 0.01, ***P < 0.001, ****P < 0.0001)

To study the biocompatibility of composite hydrogels, in the first step, rat cardiomyocytes H9C2 were cultured on CS–Gel and CS–Gel–BP@PDA. CCK-8 cell activity assay showed that the relative cell activity of CS–Gel was 73.0% and that of CS–Gel–BP@PDA was 81.9%, and there was no significant difference between the 2 groups (Fig. 4A). Further, we stained the live cells green with calcein-AM, and the fluorescence images showed that the cells in each group grew well (Fig. 4B). These results showed that all hydrogels had good biocompatibility. Their loose and porous structure and the wrinkled structure of BPNSs provided a place for cell growth.

Next, DHE and DCFH-DA staining were used to examine the scavenging effect of the CS–Gel and CS–Gel–BP@PDA composite hydrogels on intracellular ROS levels (Fig. 4C and D). In DHE and DCFH-DA fluorescence detection results, red fluorescence and green fluorescence were the most intense in the H_2_O_2_ group, which represented the presence of a large amount of ROS in H9C2 cells. The fluorescence intensity of the CS–Gel hydrogel group was reduced, and the fluorescence intensity of H9C2 cells incubated with the CS–Gel–BP@PDA composite hydrogel was significantly decreased. This indicated that CS–Gel–BP@PDA could effectively inhibit the oxidative stress of cells. Quantitative analysis showed that in the 2 staining results, the fluorescence intensity of the CS–Gel–BP@PDA composite hydrogel group was slightly higher than that of the control group, which was negligible compared with the H_2_O_2_ group. Compared with the H_2_O_2_ group, the CS-Gel-BP@PDA composite hydrogel group showed 57.3% and 55.2% higher ROS clearance, respectively.

Mitochondrial membrane potential (MMP) is one of the indicators to predict early apoptosis, and the decrease in MMP marks the occurrence of apoptosis. We used JC-1 MMP assay kit to evaluate MMP in H9C2 cells in each treatment group (Fig. 4E). Compared with the H_2_O_2_ group, the red fluorescence of the CS–Gel, and CS–Gel–BP@PDA group was stronger. H9C2 cells with H_2_O_2_ exhibited the strongest green fluorescence, while this damage was importantly improved after treatment with CS–Gel–BP@PDA. The results of the JC-1 assay demonstrated that CS–Gel–BP@PDA composite hydrogel could reduce ROS-induced mitochondrial damage.

### CS-Gel-BP@PDA anti-inflammatory activity in vitro

After MI, a large number of ROS are produced, and a large number of proinflammatory factors accompanying them induce macrophages to polarize into the M1 phenotype, which leads to an intensified inflammatory response in the infarct area. Therefore, anti-inflammatory treatment in the early stage of MI is particularly important, which is an effective way to prevent further death of the heart muscle.

**Fig. 5.**
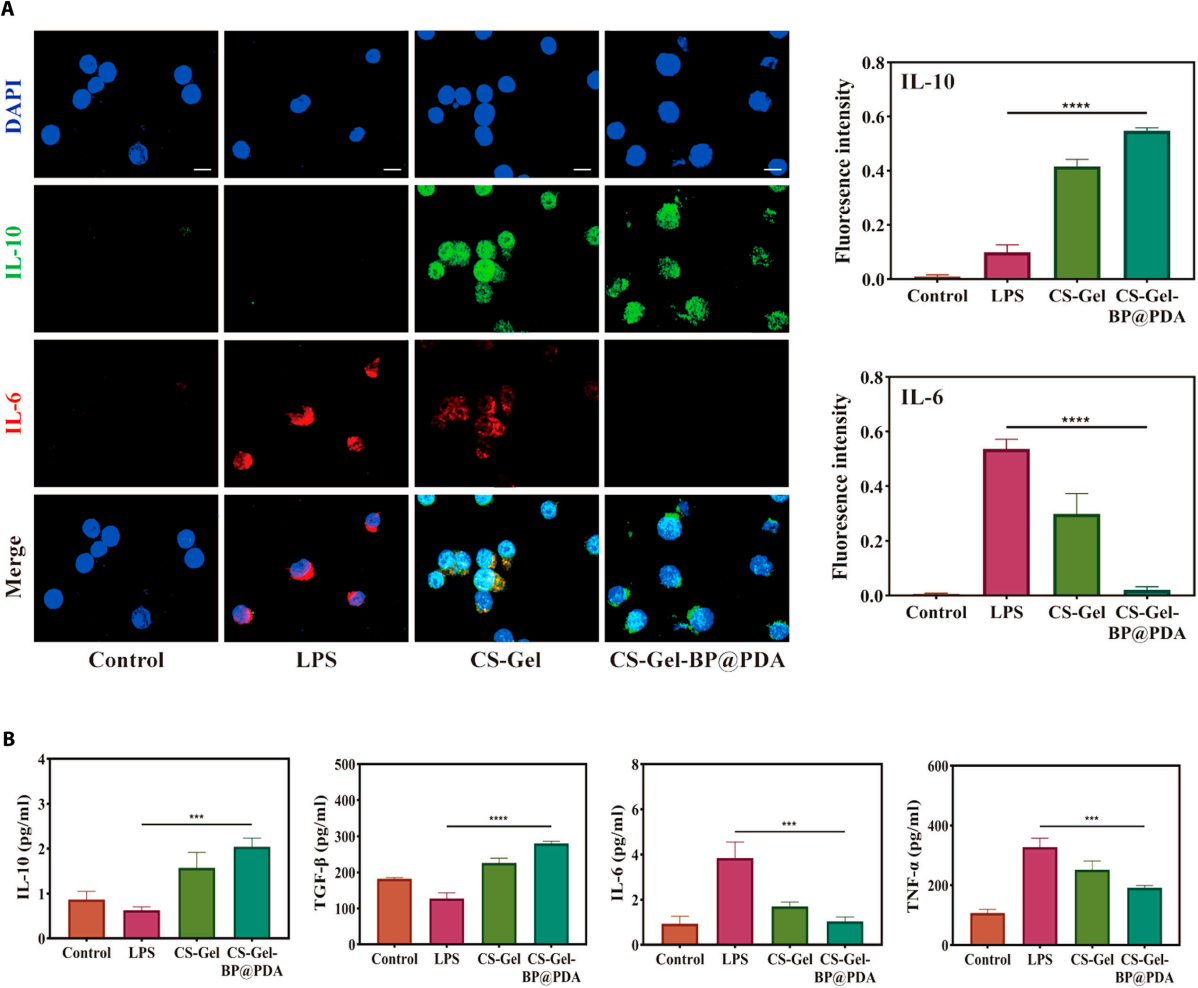
The anti-inflammatory ability of composite hydrogel in vitro. (A) Immunofluorescence colocalization and quantitative statistical analysis of IL-10 and IL-6 in different treatment groups. (B) ELISA results of IL-6, IL-10, TNF-α, and TGF-β in the supernatant of RAW246.7 cell culture medium in different treatment groups. (Scale bar: 10 µm. *P < 0.05, **P < 0.01, ***P < 0.001, ****P < 0.0001) DAPI, 4′,6-diamidino-2-phenylindole.

We detected the anti-inflammatory activity of CS-Gel-BP@PDA complex hydrogel using RAW264.7, induced macrophage M1 phenotype polarization by lipopolysaccharides (LPS) and treated with CS-Gel or CS-Gel-BP@PDA. Immunofluorescence colocalization of IL-6 and IL-10 showed that the expression of inflammatory IL-6 was significantly reduced in the CS-Gel-BP@PDA treatment group, suggesting that CS-Gel-BP@PDA effectively promoted the polarization of M1 phenotype macrophages into M2 phenotype. A similar effect was observed in the CS-Gel treatment group (Fig. 5A). In addition, the supernatant of RAW264.7 cell culture medium in different treatment groups was collected and the expressions of IL-6, IL-10, TNF-α, and TGF-β were detected by enzyme-linked immunosorbent assay (ELISA) (Fig. 5B). The expression levels of IL-6 and TNF-α in M1 phenotype macrophages were significantly increased in the LPS group, while the expression levels of IL-10 and TGF-β in M2 phenotype macrophages were highest in CS-Gel-BP@PDA group. The results showed that CS-Gel-BP@PDA and CS-Gel treatment significantly decreased the polarization of M1 phenotype macrophages and, in turn, promoted the polarization of M2 phenotype macrophages toward anti-inflammatory macrophages.

### CS–Gel–BP@PDA improves cardiac function and reduces myocardial fibrosis in rats with MI

**Fig. 6.**
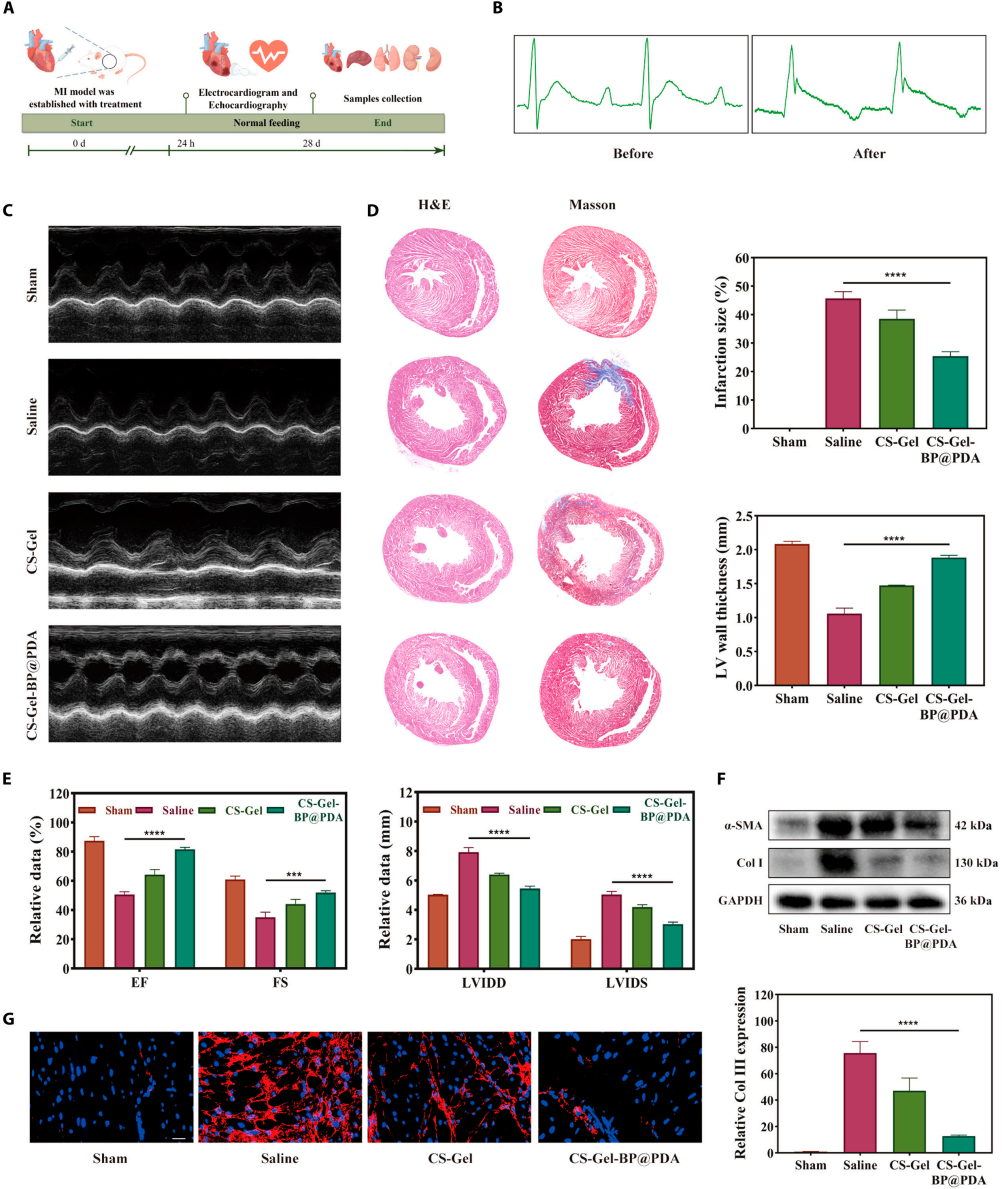
CS–Gel–BP@PDA improved cardiac function and myocardial fibrosis in MI rats. (A) Timeline of experiments in rats treated with CS–Gel–BP@PDA composite hydrogel. (B) Electrocardiograms of rats before and after MI. (C) Echocardiography and (D) H&E, Masson staining, and quantitative statistics of cardiac sections in different treatment groups on day 28 after MI. (E) Quantitative analysis of EF, FS, LVIDD, and LVIDS in different treatment groups. (F) WB detection of α-SMA and Col I protein expression at infarct sites in different treatment groups. (G) Col III immunofluorescence staining and quantitative statistical analysis of infarct sites in different treatment groups. (*P < 0.05, **P < 0.01, ***P < 0.001, ****P< 0.0001) GAPDH, glyceraldehyde-3-phosphate dehydrogenase.

The rat MI model was established by surgical ligation of the left anterior descending coronary artery (Fig. 6A). Electrocardiogram monitoring was performed before and after the operation, and the ST segment was importantly elevated after the operation, which proved that the model was successfully established (Fig. 6B).

After MI, proinflammatory signals such as Toll-like receptors and IL-1 induce an inflammatory response, and neutrophils and monocytes infiltrate the infarct site, leading to cardiomyocyte death. The inflammatory response abates after about 7 d, at which point fibroblasts promote collagen synthesis and scar tissue is formed to repair the infarct site. After 28 d, the structural matrix protein network is formed in the infarct tissue, and the scar gradually matures [30,31]. Therefore, echocardiography and tissue section staining were used to evaluate the therapeutic effect of CS–Gel–BP@PDA composite hydrogel 28 d after the MI rat model was constructed (Fig. 6C). Compared with the saline group, the CS–Gel–BP@PDA treatment group displayed significant improvement in cardiac function. After 4 weeks of treatment, the CS–Gel–BP@PDA-treated group showed significant improvement in ejection fraction (EF) and fractional shortening (FS). Furthermore, CS–Gel–BP@PDA treatment reduced LV internal dimension diastole (LVIDD) and internal dimension systole (LVIDS) (Fig. 6E). The results showed that CS–Gel–BP@PDA sustained and effective treatment rescued cardiac function after MI.

H&E and Masson staining were used to assess collagen deposition and identify areas of fibrosis (Fig. 6D). Significant fibrosis formation and a large area of blue collagen deposition could be observed in the saline group. Compared with the saline group, there was no obvious fibrosis and collagen deposition in the CS–Gel–BP@PDA treatment group, and the CS–Gel–BP@PDA group had a smaller infarct size and the largest LV wall thickness. Subsequently, we verified the expression of fibrosis protein α-smooth muscle actin (α-SMA) and collagen I (Col I) by Western blot (WB) assay (Fig. 6F and Fig. S7). α-SMA and Col I were abundantly expressed in the saline group, and after treatment with composite hydrogel CS-Gel-BP@PDA, fibrotic protein expression was significantly reduced. In addition, immunofluorescence staining of collagen III (Col III) showed that Col III fluorescence intensity was weakest at the infarct site in the composite hydrogel CS-Gel-BP@PDA group (Fig. 6G). Based on this, we concluded that CS-Gel-BP@PDA could effectively inhibit the progression of postinfarction myocardial fibrosis. Therefore, the intervention of CS-Gel-BP@PDA is of great significance for the improvement of cardiac function or the protection of cardiac structure after infarction.

### CS–Gel–BP@PDA inhibits cardiomyocyte apoptosis by antioxidative stress

**Fig. 7.**
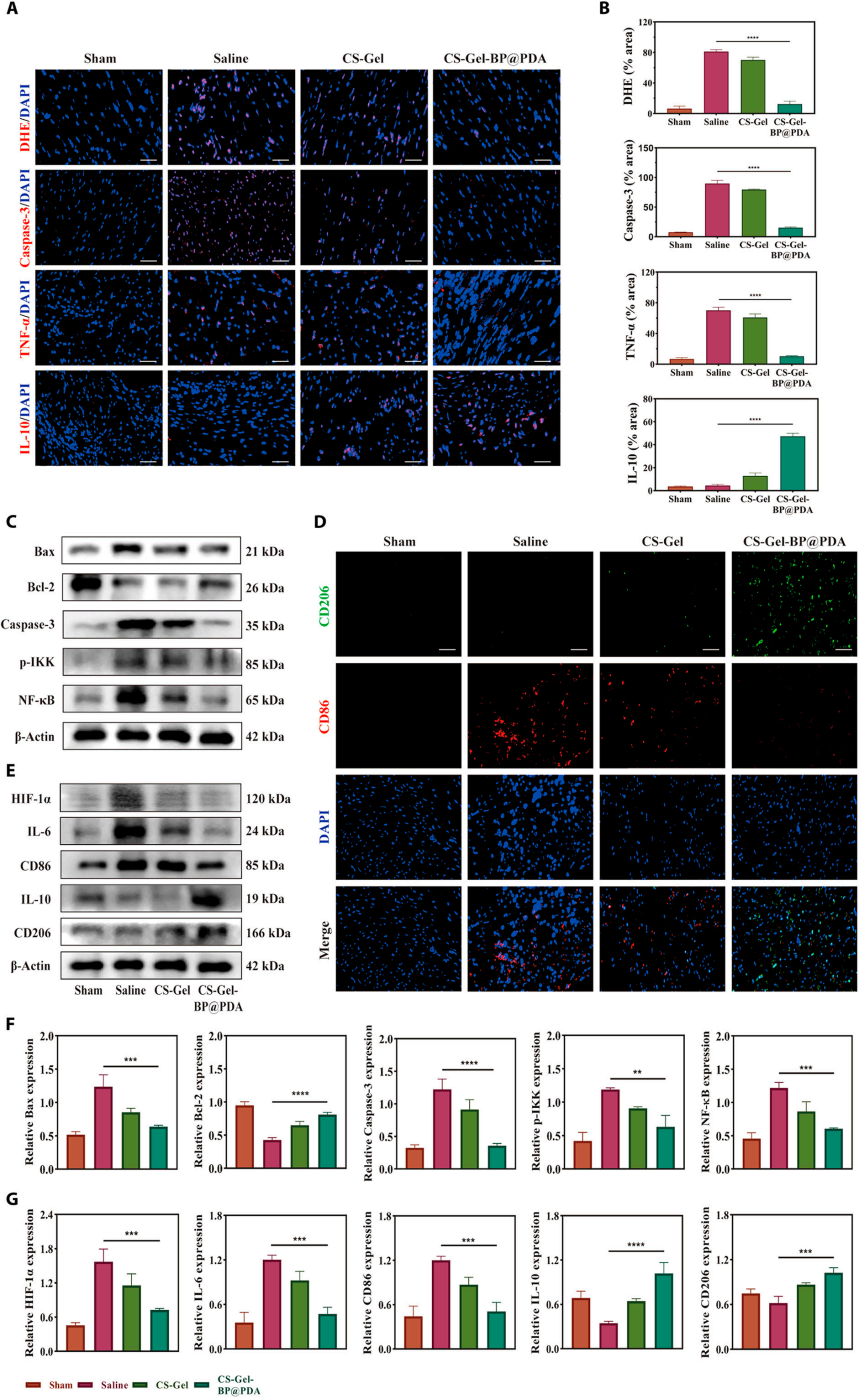
CS–Gel–BP@PDA removal of ROS in the infarcted microenvironment of rats with MI alleviated oxidative stress and promoted macrophage polarization to M2 phenotype. (A) Immunofluorescence staining of DHE, Caspase-3, TNF-α, and IL-10 at the infarct site after 28 d of modeling in each group. (B) Quantitative statistical analysis of DHE, caspase-3, TNF-α, and IL-10 immunofluorescence staining at the infarct site on day 28 of modeling in each group. (C) WB detection of NF-κB apoptosis pathway-related protein expression and (F) quantitative statistical analysis on day 28 of modeling in each group. (D) Immunofluorescence co-localization staining of CD206/CD86 in the infarcts on day 28 of modeling in each group. (E) WB detection of macrophage polarization-related protein expression and (G) quantitative statistical analysis on day 28 of modeling in each group. (Scale bar: 50 µm. *P < 0.05, **P < 0.01, ***P < 0.001, ****P < 0.0001)

The generation of ROS at the infarct site can trigger an inflammatory response and cause cardiomyocyte apoptosis, so early and effective ROS clearance can effectively improve the prognostic progression of infarction. As mentioned previously, CS-Gel-BP@PDA has excellent sustained ROS removal ability; given this, we first detected the ROS content in myocardial tissues in the infarcted area by DHE staining, and the results showed that the ROS content in myocardial tissues in the CS-Gel-BP@PDA group was significantly reduced, suggesting that CS-Gel-BP@PDA was able to effectively remove ROS (Fig. 7A and B). To further determine the level of oxidative stress, we examined the content of superoxide dismutase and malondialdehyde in the infarcted tissues by ELISA. The results showed that the composite hydrogel CS-Gel-BP@PDA significantly reduced the levels of oxidative stress enzymes in the infarcted site compared with the saline group. The superoxide dismutase activity of CS-Gel-BP@PDA increased from 37.03 to 75.43 U/mg prot in the saline group, whereas the malondialdehyde content increased from 19.29 to 12.98 nmol/mg prot (Fig. S8). As we know, oxidative stress induces the occurrence of a series of subsequent inflammatory response chains; whether the effective scavenging of ROS by CS-Gel-BP@PDA could inhibit the occurrence of inflammatory responses, we detected the apoptotic protein expression and specific inflammatory factor levels in the infarcted area using immunofluorescence staining (Fig. 7A and B). Both apoptotic protein levels and TNF-α proinflammatory factor levels were high in the saline group, and significant fluorescent signals were detected. Notably, the inflammatory response was also attenuated in the CS-Gel-treated group, which may be due to the role of the anti-inflammatory effect of CS-Gel itself. After CS-Gel-BP@PDA composite hydrogel treatment, the expression of IL-10, which inhibits inflammation, was elevated, whereas ROS levels, apoptotic protein levels, and TNF-α proinflammatory factor expression were significantly reduced.

To further verify the protective effect of the composite hydrogel on myocardial cell apoptosis, the expression of NF-κB-related proteins in the classical apoptotic pathway was verified by WB (Fig. 7C and F). The results revealed that the expressions of proapoptotic proteins Bax and caspase-3 were the highest in the saline group, while they were significantly decreased in the CS–Gel–BP@PDA group. Contrarily, Bcl-2, which inhibits apoptosis, was more expressed in the CS–Gel–BP@PDA group. In addition, both p-IKK and NF-κB were abundantly expressed in the saline group. These results indicate that orthotopic injection of CS–Gel–BP@PDA composite hydrogel can significantly reduce ROS levels, inhibit myocardial cell apoptosis, and alleviate the damage caused by ischemia–hypoxia in rats with MI, which confirmed the potential therapeutic effect of CS–Gel–BP@PDA composite hydrogel on improving myocardial viability.

### CS–Gel–BP@PDA improved the inflammatory response in the infarct area by promoting macrophage polarization to the M2 phenotype

ROS-induced inflammation disrupts the myocardial microenvironment. In this process, M1 phenotype macrophages play a proinflammatory role, leading to massive inflammatory infiltration at the infarct site. However, M2 phenotype macrophages are cells that suppress inflammation. Therefore, we examined the phenotype of macrophages at the infarct site by CD206/CD86 immunofluorescence colocalization (Fig. 7D). Fluorescence images showed that a large number of red-labeled CD86-positive cells were aggregated in the saline groups, while green CD206-positive cells were rarely observed. Compared with the saline group, the number of CD206-positive cells was significantly increased in the CS–Gel–BP@PDA treatment group (Fig. S9).

Next, we further examined the expression of macrophage-related proteins in the infarcted tissue by WB (Fig. 7E and G). IL-6 and CD86 expressed by M1 phenotype macrophages were highly expressed in the saline groups but were significantly reduced in the CS–Gel–BP@PDA treatment group. On the contrary, the expressions of IL-10 and CD206, which represent anti-inflammatory M2 phenotype macrophages, were significantly increased in the CS–Gel–BP@PDA treatment group. Some studies have reported that hypoxia-inducible factor-1α (HIF-1α) plays an important role in M2 phenotype macrophage polarization [32]. To explore the mechanism by which composite hydrogels promote macrophage M2 phenotype polarization, we examined the expression of HIF-1α. In the saline group, HIF-1α expression was up-regulated and significantly decreased after treatment with CS-Gel-BP@PDA. Combined with the previous experimental results, CS-Gel-BP@PDA promotes the decrease of HIF-1α and affects the HIF-1α/NF-κB inflammatory signaling cascade, which promotes macrophage M2 phenotype polarization. The above results further demonstrated that the treatment with CS–Gel–BP@PDA composite hydrogel can promote the polarization of macrophages to the M2 phenotype that suppresses inflammation, thereby achieving targeted resolution of inflammation.

### Implantation of CS–Gel and CS–Gel–BP@PDA did not cause damage to other organs of the rats

**Fig. 8.**
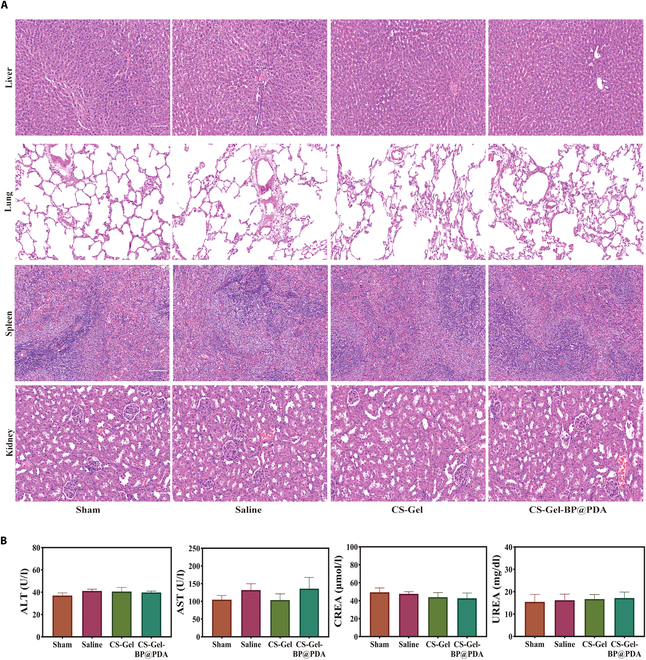
System toxicity. (A) Systemic toxicity evaluation. H&E staining of liver, lung, spleen, and kidney in Sham, Saline, CS-Gel, and CS-Gel-BP@PDA groups on day 28 after modeling. (B) Liver and kidney function (ALT, alanine aminotransferase; AST, aspartate aminotransferase; CREA, creatinine; UREA, urea) was evaluated in Sham, Saline, CS-Gel, and CS-Gel-BP@PDA groups on day 28 after modeling.

Biosafety is a prerequisite for the application of biomaterials in the field of medicine; because of this, we conducted relevant tests on the biosafety of the composite gel. First, we examined the tissue structure of the liver, spleen, lung, and kidney by H&E staining in Sprague–Dawley rats and the structure showed that no obvious inflammatory infiltration or tissue structural changes were found in each organ (Fig. 8A). The results of serum liver and kidney function showed that there was no significant difference in liver and kidney function among the 4 groups (Fig. 8B). The above results demonstrated that the composite hydrogel had excellent biosafety and that myocardial in situ implantation did not cause any damage to the remaining organs 28 d after implantation.

## Discussion

MI is one of the acute syndromes with high morbidity and mortality. Sustained oxidative stress and inflammatory response are the main causative factors leading to the malignant prognosis of MI. In this study, we designed a CS-Gel-BP@PDA composite hydrogel with CS-Gel as a carrier to achieve in situ palliative release of BPNSs in the infarct region. Our results demonstrated that this composite hydrogel system effectively achieved the scavenging of excessive ROS in the infarct region, attenuated oxidative stress, promoted macrophage M2 phenotypic polarization, reduced inflammatory responses, improved the microenvironment, and facilitated the recovery of cardiac function in rats by sustained brady release of BPNSs.

Macrophages are the largest subpopulation of immune cells in the heart and exhibit excellent plasticity and flexibility during lesion progression [33]. In the early stage of MI, macrophages are recruited to the infarcted area and are predominantly of the M1 phenotype, exerting a proinflammatory effect. As healing advances, macrophages shift from the M1 phenotype to the M2 phenotype, which participates in restricting and inhibiting inflammatory responses and promoting tissue repair [34]. Therefore, timely promotion of macrophage M2 phenotype polarization is important for the prognosis of infarction. However, the release of excessive mitochondrial ROS from damaged myocardial cells after MI is in turn a major factor contributing to the sustained M1 phenotypic polarization of macrophages, and thus, the sustained clearance of intracellular ROS from damaged cells is essential for the improvement of inflammatory response by M2 phenotypic polarization of macrophages in the late phase of MI. As mentioned previously, BPNSs have excellent ROS-responsive activity and are capable of scavenging ROS with high efficiency; therefore, the purpose of sustained slow release of BPNSs was achieved by doping BPNSs within CS-Gel and utilizing the biologically degradable properties of the gel with the aim of being able to achieve optimal therapeutic efficacy in MI. Our study demonstrated that CS-Gel-BP@PDA significantly cleared ROS from tissues in the infarcted area, importantly improved mitochondrial function, and promoted macrophage polarization toward the M2 phenotype. Further, we explored the mechanisms by which it manipulates macrophage polarization.HIF-1α is a heterodimeric transcription factor that is normally degraded by prolyl-4-hydroxylase hydroxylation under normoxic conditions. However, elevated ROS levels in oxidative stress environments lead to inactivation of prolyl-4-hydroxylase and greater stabilization of HIF-1α. This stabilizing effect contributes to the translocation of HIF-1α into the nucleus to activate the transcription of hypoxia-responsive glycolytic genes, including PDK1 and Glut1 [35]. Studies have shown that M1 macrophages are highly dependent on glycolytic metabolism, whereas M2 macrophages are mainly dependent on oxidative phosphorylation [36]. Therefore, CS-Gel-BP@PDA may induce macrophage M2 phenotype polarization by decreasing ROS and increasing HIF-1α degradation. Our study demonstrated this hypothesis. CS-Gel-BP@PDA significantly reduced HIF-1α expression at the infarct site in MI rats, with a significant increase in macrophage markers of the M2 phenotype. In addition, decreased HIF-1a expression within macrophages further attenuated the inflammatory response, possibly by limiting activation of the ROS/HIF-1α/NF-κB pathway.

MI injury activates cardiac fibroblasts and forms non-contractile scar tissue. Traditional treatment modalities face great challenges such as irreversible myocardial necrosis and decreased cardiac function. Existing clinical treatment modalities mainly include interventional and pharmacologic therapies [37]. Percutaneous coronary intervention is less invasive and has a high success rate of revascularization. However, the bleeding rate associated with its surgical access reaches 30% to 70%. Vessel perforation, hypotension, malignant arrhythmias, and acute kidney injury are also serious complications of percutaneous coronary intervention. Studies have shown a 17% reduction in early mortality with pharmacologic treatment within the first 2 h of MI symptom onset. However, pharmacotherapy is poorly targeted, prone to cerebral hemorrhage, and its clinical management is more complex. In recent years, cardiac regeneration strategies to treat MI have gradually come into the limelight. However, its efficacy still needs to be improved due to the poor self-regeneration ability of cardiomyocytes. Stem cell implantation and gene therapy, which utilize stem cells or genes to promote cardiac regeneration, are complicated to prepare, have low cell survival and retention rates, and have low gene transfection efficiency. In addition, the efficacy of treatments, including drug and cell therapies, is greatly limited by the cardiac microenvironment, which may lead to cardiac remodeling after MI and result in heart failure [38]. We designed CS-Gel-BP@PDA composite hydrogel to successfully rescue cardiac function by modulating oxidative stress and inflammatory responses in the MI microenvironment without stem cells, drugs, or genes. Clinical studies have shown that patients treated after MI have the highest 6-month survival rate with EF greater than 50%, and infarct size less than 20% favors long-term survival [39,40]. In our study, postoperative echocardiography well reflected the improvement in cardiac function. EF, FS, LVIDD, and LVIDS were significantly improved. EF reached 81.6%, and infarct size was reduced to 25.4%. In contrast to colchicine, which may induce pneumonia when used in clinical therapy, the composite hydrogel did not provoke marked long-term side effects.

Biosafety is a prerequisite for the application of biomaterials. In our study, all the components of CS-Gel-BP@PDA had natural degradation properties. The degradation products of BPNSs were phosphates that were harmless to human body, and both CS and Gel were natural components extracted from living organisms, and their degradation products were low-antigenicity and nontoxic products. Our results showed that CS-Gel-BP@PDA did not show significant systemic toxicity after 28 d of implantation in MI rats. It has been demonstrated that CS-Gel is completely degraded after up to 16 weeks of in vivo application, and no other tissue or organ toxicity was observed [41,42]. Therefore, it is reasonable to believe that CS-Gel-BP@PDA implantation does not cause long-term side effects.

Biomaterial-based drug delivery systems need to fulfill the characteristics of less invasiveness and targeting so as to ensure their therapeutic efficiency and safety. Currently, common drug delivery systems include nanocarriers, particles, injectable hydrogels, and implantable cardiac patches [43]. Nanocarriers are administered intravenously and are less invasive, but due to their intrinsic size and mobility, it is difficult to sustainably accumulate in the region of MI, and most of them are intercepted in blood-filtering organs (liver, spleen, and kidneys) during circulation and are cleared from the body by phagocytosis or as excretory products. Unlike the burst drug release from nanocarriers, particles are capable of predictable, regulated, and sustained release of therapeutic payloads. However, solvent evaporation techniques for preparing particles are prone to losses as well as polydisperse size distributions. Injectable hydrogels deliver biologics while providing mechanical support for the MI by acting as a filler, which is an advantage that differentiates it from the delivery systems mentioned above. Injectable hydrogels mostly reach the infarct site by intramyocardial or intrapericardial in situ injections, intracoronary injections, and transendocardial injections by catheterization systems. Intramyocardial or intrapericardial in situ injections are mostly done through open-heart surgery, which is a highly invasive implantation method. The use of thoracoscopy has importantly reduced the invasiveness during the procedure, which may become the most desirable implantation modality for injectable hydrogels [44]. Intracoronary injections and transendocardial injections with catheter systems are less invasive. However, the former may cause thrombosis, and the latter is prone to the surgical complication of pericardial effusion. In contrast to the other delivery systems, implantable cardiac patches are the most invasive application, with postoperative complications including thoracic adhesions, secondary cardiac injury, and patch dislodgement. In our study, the injectable CS-Gel-BP@PDA composite hydrogel was administered by intramyocardial injection, which was simple and fast to deliver the drug precisely to the infarct site. This has the advantage of being both less invasive and enabling targeted drug delivery. Such a mode of drug delivery may allow for minimally invasive treatment in the clinic.

However, hydrogel-based therapies still face challenges. Our study used open-heart surgery to construct a model of MI while hydrogels were injected in situ within the myocardium. In the case of spontaneous MI, open-heart surgical implantation of hydrogel is clearly not suitable for routine clinical treatment. Transendocardial injection of hydrogel via a catheterization system and thoracoscopic surgical implantation have shed light on clinical applications [45,46]. In terms of injection dosage, more clinical studies have discussed the therapeutic role of hydrogels in infarction, and they have used single injection dosages of 0.25 to 2 ml [47,48]. The gelation time after implantation also needs to be fully considered to avoid damage to the surrounding tissues. Recent studies have found that environmentally responsive hydrogels may be a better choice, as they are able to undergo state transitions in response to specific stimuli, e.g., temperature, pH [49,50]. Overall, the composite hydrogels developed in this study achieved sustained clearance of overloaded ROS in the infarcted region by slow release of BPNSs, attenuated oxidative stress, promoted macrophage M2 phenotypic polarization and thus inhibited inflammatory response, and improved cardiac function and myocardial fibrosis progression after MI, which is a promising therapeutic strategy.

Here, we designed an injectable composite hydrogel CS–Gel–BP@PDA with the time-released capability to achieve sustained release of therapeutic nanomedicine at the lesion site through in situ drug delivery at the MI site. Our results showed that continuous time-released BP@PDA could effectively eliminate excessive ROS in the MI microenvironment, improve MMP, and play an antioxidative stress role. Interestingly, the composite nanogel could also effectively inhibit the occurrence of postinfarction inflammatory storm by inhibiting the HIF-1α/NF-κB inflammatory signaling cascade, promoting macrophage polarization to the M2 phenotype, and switching from proinflammatory to anti-inflammatory, thus preventing cardiomyocyte apoptosis to effectively inhibit the occurrence of adverse infarction progression. Consequently, the use of CS–Gel–BP@PDA composite hydrogel to effectively treat MI and improve adverse events after MI is of great significance for the future clinical treatment of MI.

## Ethical Approval

All animal experiments were conducted according to the protocol approved by the Institutional Animal Care and Use Committee, Huazhong University of Science and Technology (IACUC Number: 3440).

## Data Availability

The datasets used and/or analyzed during the current study are available from the corresponding author upon reasonable request.
