# Supplementary material for: Time-Released Black Phosphorus Hydrogel Accelerates Myocardial Repairing through Antioxidant and Motivates Macrophage Polarization Properties
Source: Biomater Res. 2024 May 8;28:0029. doi: 10.34133/bmr.0029 (PMC11077294; doi:10.34133/bmr.0029)
Supplement: Supplementary 1 — Figs. S1 to S9 Movie S1 [file bmr.0029.f1.zip › Supplemental Material.docx]

Supporting Information

Time-released Black Phosphorus Hydrogel Accelerates Myocardial Repairing through Antioxidant and Motivate Macrophage Polarization Properties

*Jiahui Zhang^1^, Di Sun^1^, Yuhan Liao^1^, Bingxin Cao, Ran Gao, Zhuanglin Zeng, Chuansheng Zheng *, Yumiao Wei* and Xiaopeng Guo**

**Total Supporting Movie (S1)**

**Movie S1.** Injectable ability of composite hydrogels.

**Total Supporting Figures (S1~S9)**

**Figure S1.** Compression modulus and tensile modulus of CS-Gel and CS-Gel-BP@PDA.

**Figure S2.** Digital photographs of 0h and 24h swelling of CS-Gel, and CS-Gel-BP@PDA.

**Figure S3.** Digital photographs of natural degradation of CS-Gel-BP@PDA in vitro.

**Figure S4.** Digital photographs of degradation of BP@PDA in H_2_O_2_.

**Figure S5.** Digital photographs of the injectability of CS-Gel-BP@PDA.

**Figure S6.** Digital photographs of the adhesive ability of CS-Gel-BP@PDA.

**Figure S7.** Quantitative statistical results of α-SMA and Col I in myocardial infarction tissues of Sham, Saline, CS-Gel, and CS-Gel-BP@PDA.

**Figure S8.** Detection results of SOD and MDA in myocardial infarction tissues of Sham, Saline, CS-Gel, and CS-Gel-BP@PDA.

**Figure S9.** CD206/CD86 immunofluorescence co-localization staining quantitative statistical analysis.

**Movie S1.** Injectable ability of composite hydrogels.

**
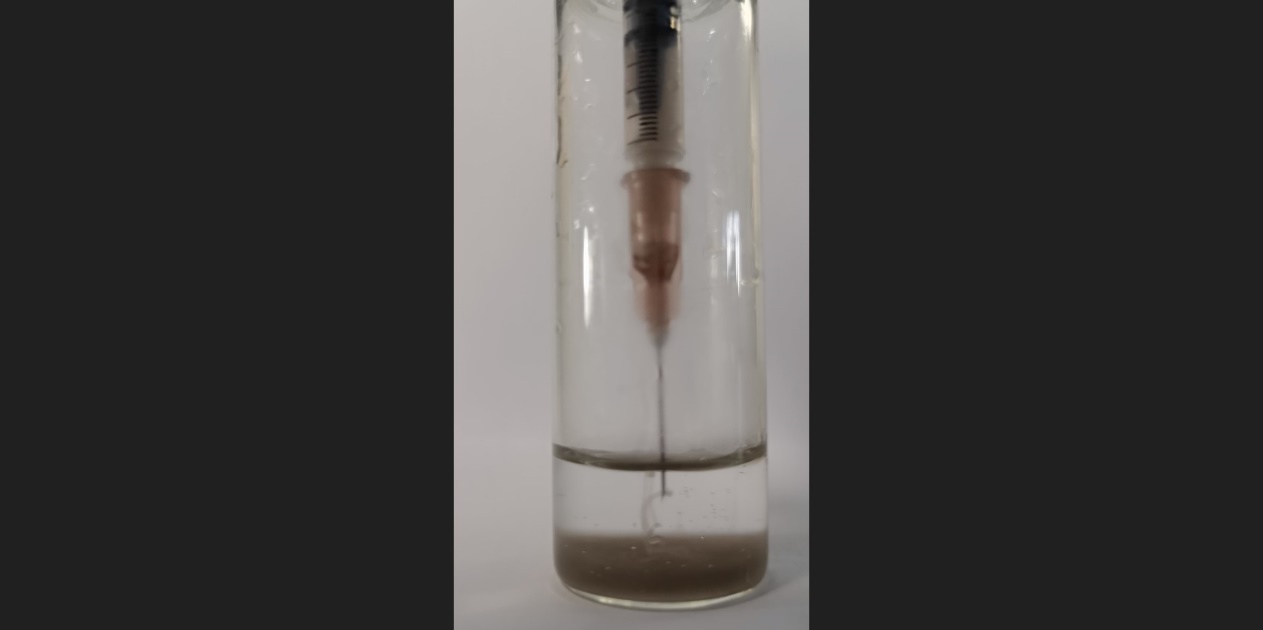
**


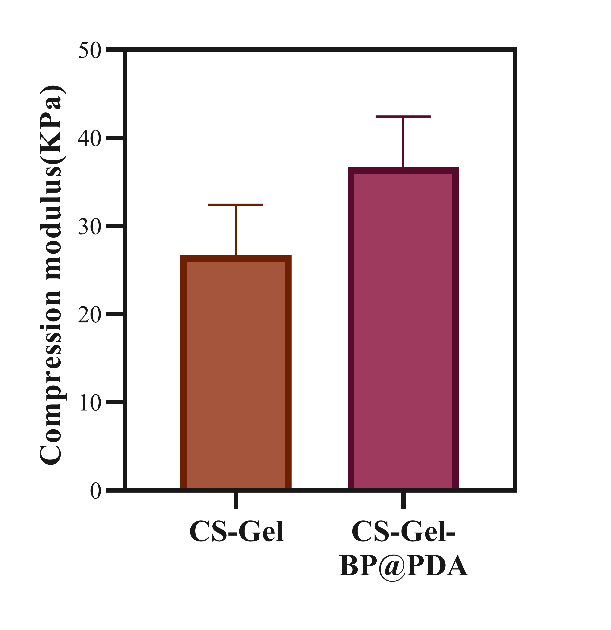

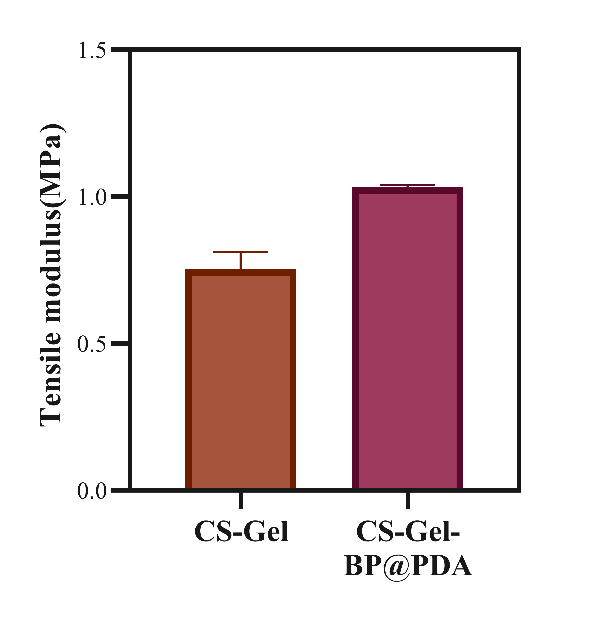


**Figure S1.** Compression modulus and tensile modulus of CS-Gel and CS-Gel-BP@PDA.


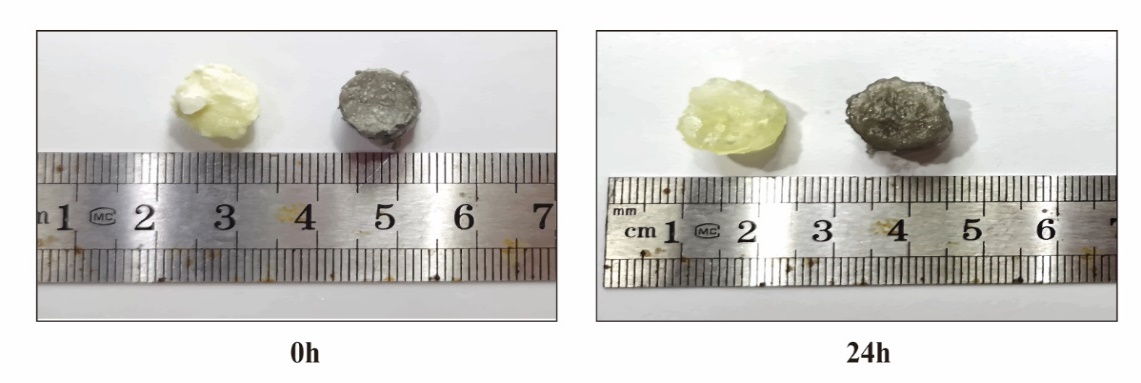


**Figure S2.** Digital photographs of 0h and 24h swelling of CS-Gel, and CS-Gel-BP@PDA.


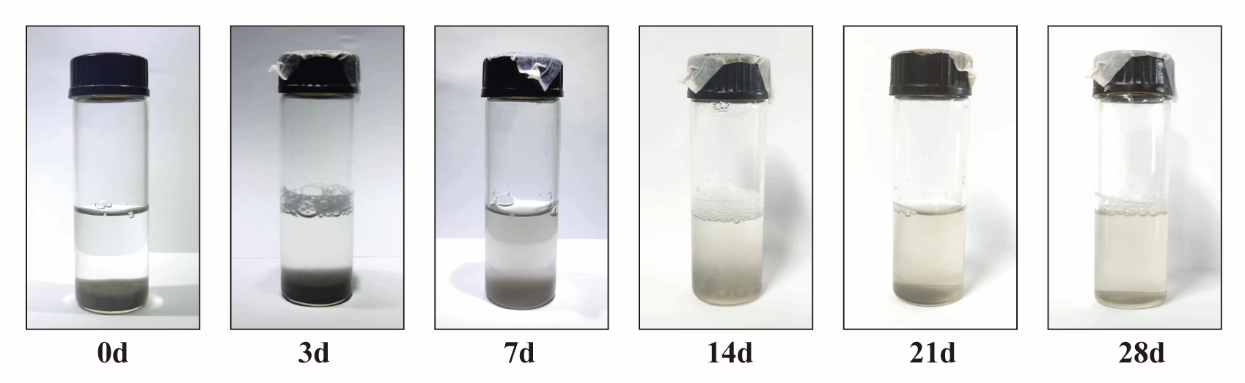


**Figure S3.** Digital photographs of natural degradation of CS-Gel-BP@PDA in vitro.


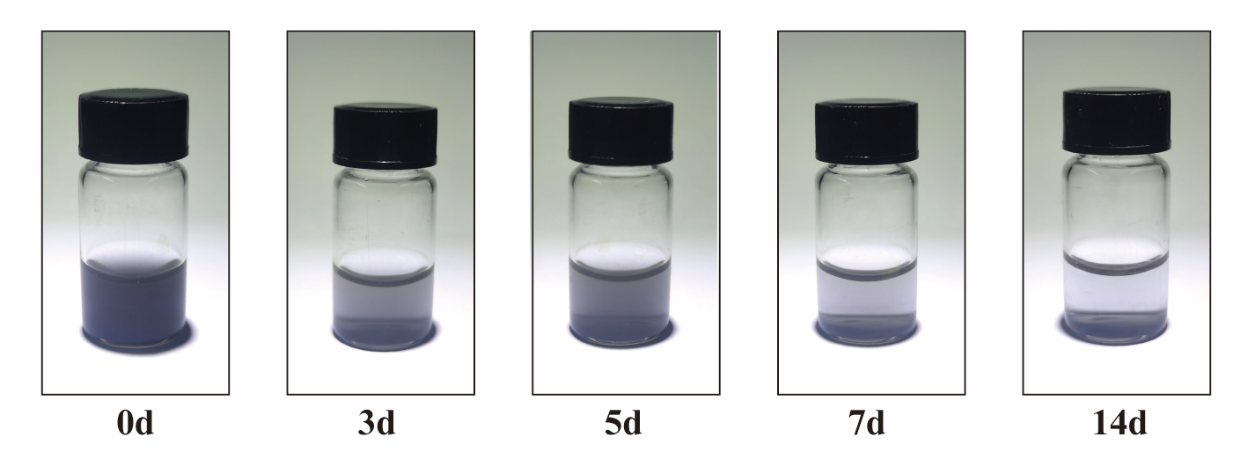


**Figure S4.** Digital photographs of degradation of BP@PDA in H_2_O_2_.


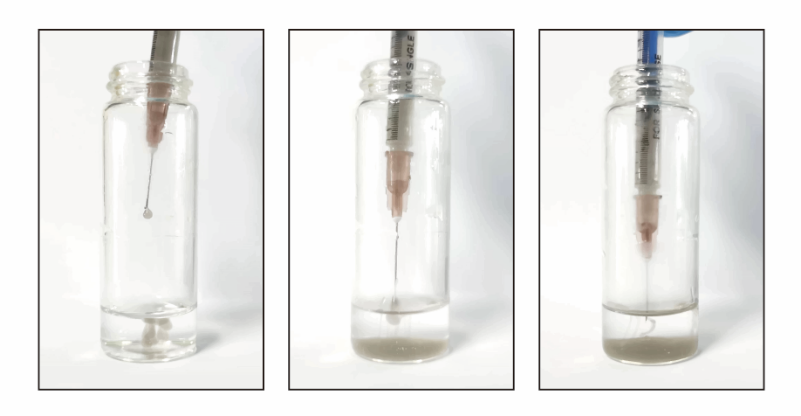


**Figure S5.** Digital photographs of the injectability of CS-Gel-BP@PDA.


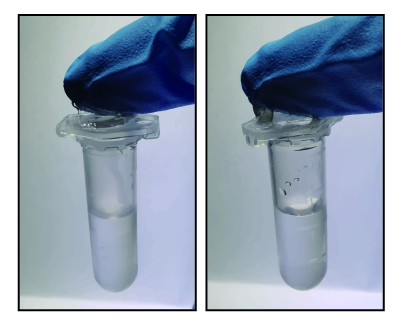


**Figure S6.** Digital photographs of the adhesive ability of CS-Gel-BP@PDA.

**
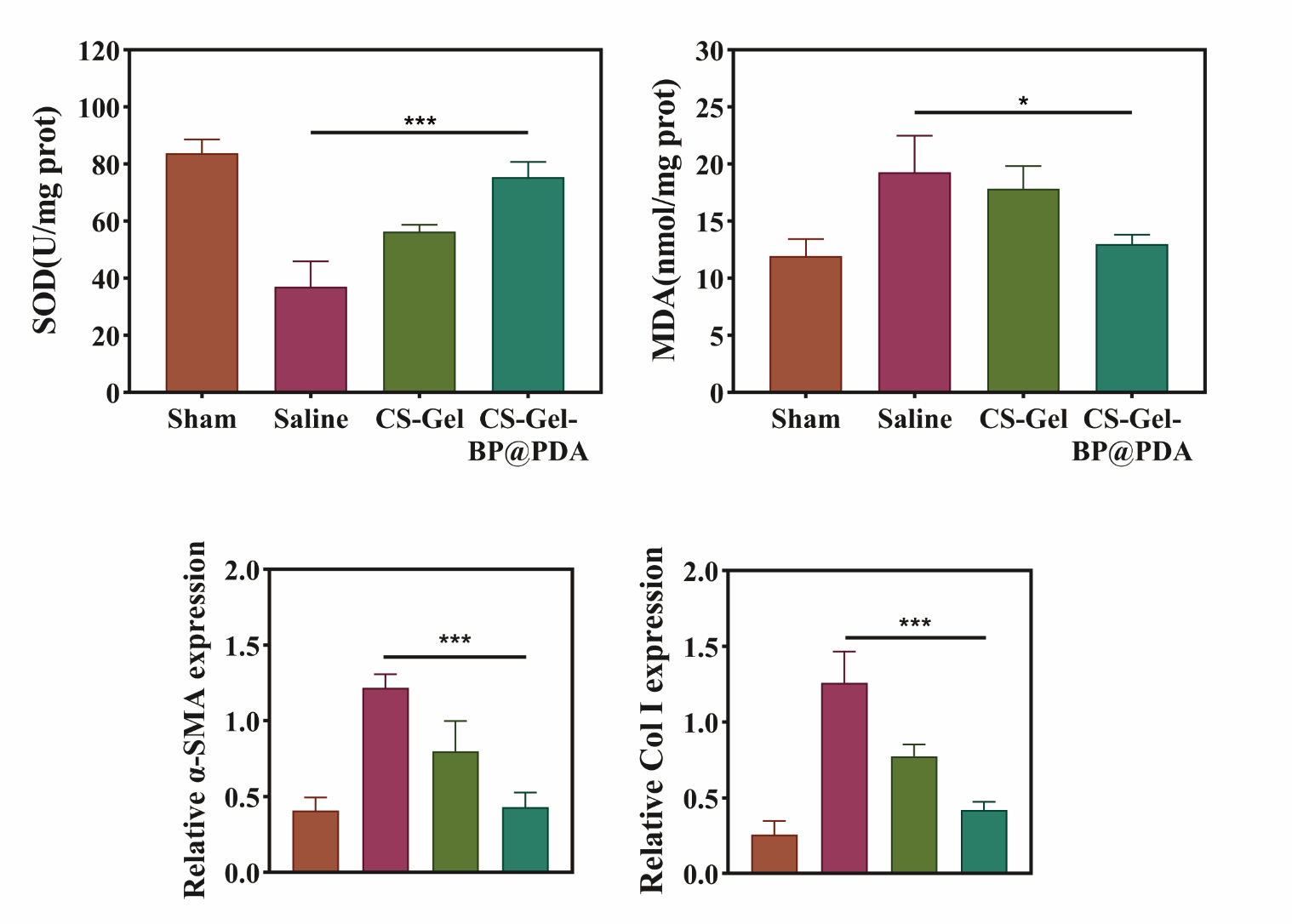
**

**Figure S7.** Quantitative statistical results of α-SMA and Col I in myocardial infarction tissues of Sham, Saline, CS-Gel, and CS-Gel-BP@PDA.

**
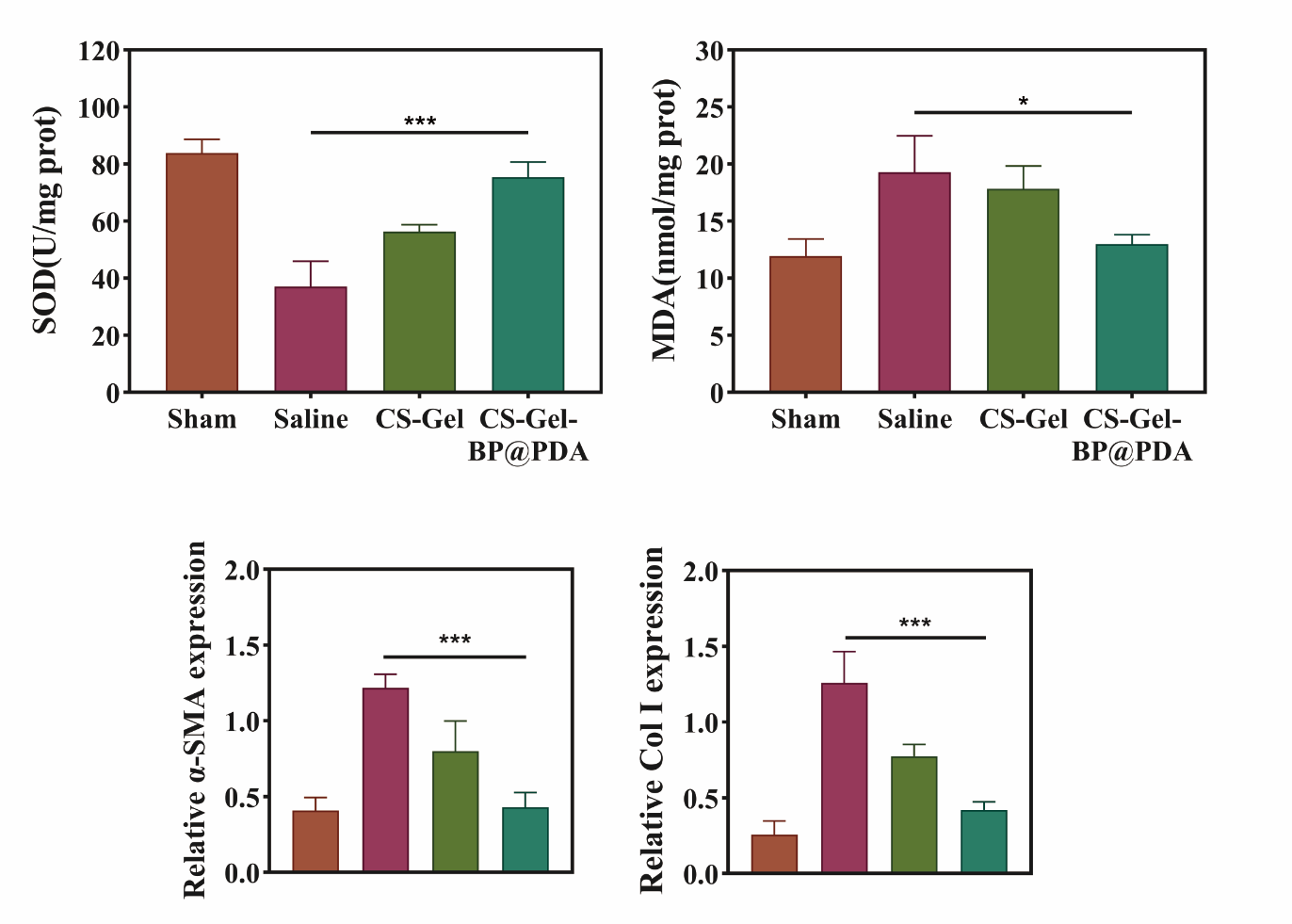
**

**Figure S8.** Detection results of SOD and MDA in myocardial infarction tissues of Sham, Saline, CS-Gel, and CS-Gel-BP@PDA.


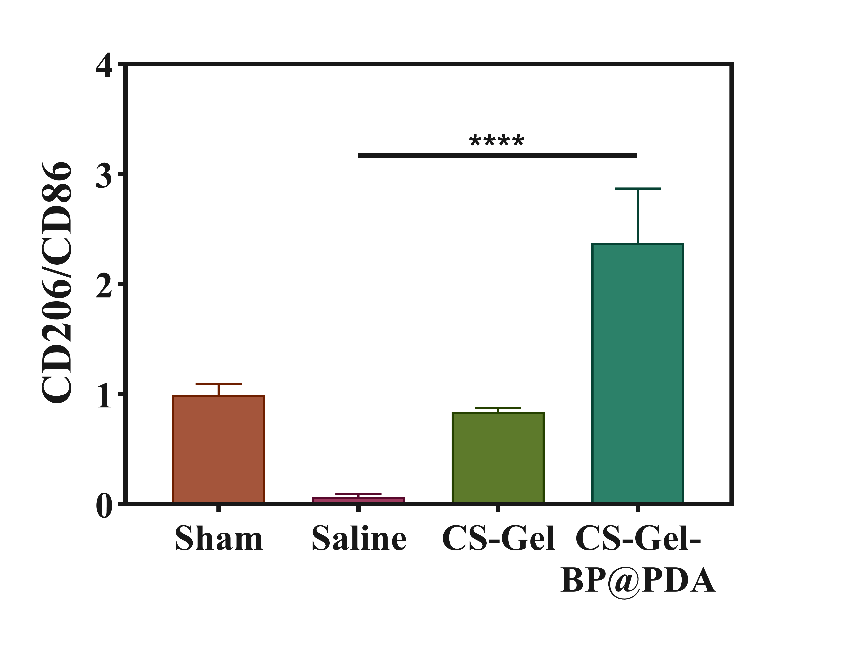


**Figure S9.** CD206/CD86 immunofluorescence co-localization staining quantitative statistical analysis.
